# Preparation of reduced graphene oxide coated flaky carbonyl iron composites and their excellent microwave absorption properties†

**DOI:** 10.1039/c7ra12984j

**Published:** 2018-01-15

**Authors:** Lihua He, Yan Zhao, Liying Xing, Pinggui Liu, Zhiyong Wang, Youwei Zhang, Ying Wang, Yunchen Du

**Affiliations:** School of Materials Science and Engineering, Beihang University Beijing 100191 China jennyzhaoyan@buaa.edu.cn +86-010-82317127 +86-010-82317127; Beijing Institute of Aeronautical Materials Beijing 100095 China; MIIT Key Laboratory of Critical Materials Technology for New Energy Conversion and Storage, School of Chemistry and Chemical Engineering, Harbin Institute of Technology Harbin 150001 China

## Abstract

In this study, a novel absorber with a high performance microwave absorption property was prepared by innovatively coupling flaky carbonyl iron (FCI) and reduced graphene oxide (rGO) nanosheets into a homogenous composite. The rGO nanosheets are tightly coated on the surface of FCI, which gives typical dielectric dispersion behavior of complex permittivity and resultantly optimizes characteristic impedance matching. Meanwhile, the introduction of rGO as a dielectric lossy material endows FCI with improved dielectric loss ability and unfading magnetic loss ability. In the frequency range of 2.0–18.0 GHz, the rGO-coated FCI composite gives a minimum value of reflection loss with −65.4 dB at 5.2 GHz when the absorber thickness is 3.87 mm and always shows effective bandwidth under −20 dB when absorber thickness is from 1.5 mm to 5 mm. The contribution of typical dielectric dispersion behavior in rGO-coated FCI is demonstrated by a delta-function method to characteristic impedance matching and the excellent microwave absorbing performance.

## Abstract 2

A absorber with frequency dispersion behavior was prepared by complete surface coating of reduced graphene oxide on flaky carbonyl iron.

## Graphical abstract

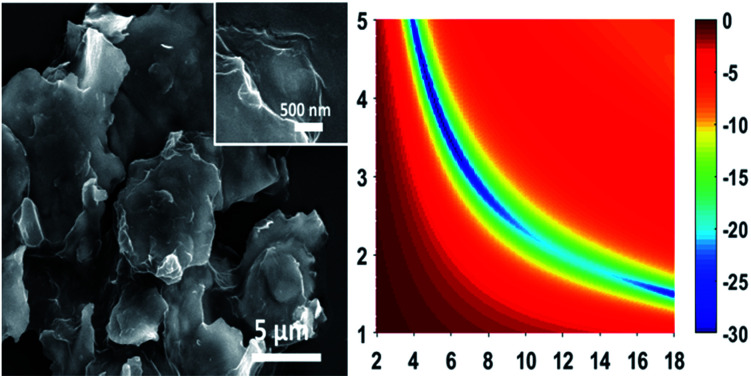

## Introduction

Microwave absorbing materials (MAMs) have sparked considerable interest in civil and military applications, which not only protect human being from electromagnetic radiation in daily life, but also exhibit stealth functionality as aircraft skin coatings. During the past decades, various MAMs have been developed based upon the demands in use, and they can be classified into two categories on the basis of their microwave loss mechanism, *i.e.*, magnetic loss materials and dielectric loss materials.^1^ Generally, magnetic loss materials include magnetic metals (Fe,^2^ Co,^3^ and Ni^4^) and their related magnetic oxides,^5,6^ whose microwave absorption performances mainly originate from hysteresis, domain wall resonance, natural ferromagnetic resonance, and eddy current effect. Dielectric loss materials normally involve carbon, SiC, BaTiO_3_ and conductive polymers materials.^7–10^ Their dielectric loss abilities mainly come from conductivity loss and polarization loss. However, these traditional MAMs frequently suffer from poor absorption and narrow bandwidth because of the limited microwave loss mechanisms. In response to this, it is widely accepted that composite materials will be qualified for the next generation of MAMs due to the synergistic and complementary effect between the individual component materials.^11–13^

Carbonyl iron as one of the magnetic metals has been considered as a very attractive candidate in microwave absorption because of its great potential in magnetic loss ability, which derives from its high saturation magnetization and magnetic permeability.^14^ Recent progress indicated that flaky carbonyl iron particles (FCI) can show smaller skin effect and higher complex permeability, and consequently exhibit superior magnetic loss ability.^15,16^ Nevertheless, carbonyl iron material is constrained by the mismatched impedance characteristic due to the large gap between its complex permittivity and complex permeability, which is extremely unfavorable to the decreasing of surface reflection to incident electromagnetic wave. It was reported that the electric-magnetic impedance match and microwave absorption performance can be improved by combining the carbonyl iron with dielectric loss materials, including MnO_2_,^17^ ZnO^18^ and polypyrrole.^19^ Although these FCI-based composites exhibit good microwave absorption properties, it has to point out that most of these improvements on microwave absorption are at the expense of the decreasing of FCI's magnetic loss ability owing to the embedding of dielectric loss material with high proportion. Besides, it is also difficult to obtain homogeneous composites because most FCI-based composites for microwave absorption were synthesized by physical mixing method, which was not beneficial to functional reproducibility and process ability. Therefore, it is still a great challenge to find an efficient synthetic strategy for preparing homogeneous FCI-based composite with remaining magnetic loss property.

Graphene has attracted tremendous attentions for its unique physical and chemical properties. These special features hold enormous potential applications in various fields, including high speed transistor,^20^ flexible display touch panel^21^ and energy storage devices^22^ to chemical sensors.^23^ Very recently, some studies have also reported that reduced graphene oxide (rGO) showed its advantages over many conventional carbon materials (*e.g.*, graphite and carbon nanotube) for microwave absorption application, which could be ascribed to its high charge carrier mobility, extraordinary electrical conductivity and residual surface defects and groups.^24–28^ However, these rGO nanosheet filled composites could not meet the demands of ideal MAM because the intrinsic high permittivity and low permeability of rGO were unfavorable to impedance matching and resulted in poor absorbing properties. Therefore, magnetic nanoparticles, including FeCo,^29^ Fe_3_O_4_,^30^ Co_3_O_4_ (ref. 31) and FeNi,^32^ have been introduced into graphene composites in order to enhance the electromagnetic wave absorption performance. The complex permeability values were improved as the magnetic nanoparticles were loaded onto graphene sheets, while the values of complex permeability were still lower compared to that of the complex permittivity. Hence, these absorbers exhibited no visible improvement in electromagnetic wave absorption performance. Recently, considerable efforts have been made to solve the problem by coupling graphene with carbonyl iron.^33–36^

Herein, a high-performance microwave absorber is prepared by innovatively chemical coupling FCI and rGO nanosheets into a homogenous composite using 3-aminopropylphosphonic acid as “link” agent, which making rGO nanosheets tightly coated the surface of FCI. The introduction of rGO can effectively improve the complex permittivity and the obtained composite exhibits the typical dielectric dispersion behavior. It is beneficial to the enhancement of dielectric loss ability and the matching of characteristic impedance. Besides, the magnetic loss ability of FCI is remained because the relative amount of coated rGO is very small. The above mentioned features are contributed to the desirable enhancement in microwave absorption. These results not only provide a novel way for preparing graphene/magnetic particles composite, but also be greatly helpful for the design and fabrication of high-performance FCI-based microwave absorbers in the future.

## Experimental

### Materials

Natural graphite flakes were purchased from Qing Dao Tian Sheng Co. Ltd (China) with an average diameter of 48 μm. 3-Aminopropylphosphonic acid (APSA) was obtained from TCI Co. Ltd. Flaky carbonyl iron (FCI) powder was supplied by Nanjing University. All the other reagents and solvents were purchased from Beijing Chemical Factory (China) and were used without further purification.

### Synthesis

#### The preparation of rGO-coated FCI

Graphene oxide (GO) was prepared from natural graphite by modified Hummers method.^37^ The as-prepared GO was first dispersed in deionized water to form suspension with concentration of 1 mg mL^−1^ by ultrasonication for 2 h. Subsequently, a certain amount of APSA was added to the above suspension under magnetic stirring. The mixture was refluxed at 95 °C for 20 h, and then dialyzed with cellulose ester dialysis membrane (MWCO is 500–1000 D) in deionized water for 7 days to remove the unreacted APSA. Finally, the mixture was dried in vacuum oven at 60 °C for 24 h and the –PO(OH)_2_ functionalized GO (GO–APSA) was prepared. The FCI powder was washed by ethanol before dispersing in ethanol/water (1 : 1, v/v). A certain amount of GO–APSA was dispersed in water by ultrasonication, and then added into the suspension of FCI powder in Ar. Subsequently, the mixture was heated to 80 °C under mechanical stirring (800 rpm). After 4 h, the suspension liquid was filtered and washed with ethanol/water (9 : 1, v/v) repeatedly to remove the unreacted GO–APSA. The obtained product was finally heated at 150 °C in vacuum oven for 24 h to get rGO-coated FCI material.

### Characterization

The microstructure of FCI particle and rGO-coated FCI particle was analyzed by field emission scanning electron microscopy (FEI, Quanta 2005), and the samples were mounted on aluminum studs using adhesive graphite tape and sputter-coated with gold before analysis. The structural information of the samples was identified by X-ray diffractometer (XRD, Rigaku D/MAXRC) using Cu Kα radiation source (*λ* = 1.5406 Å). Raman spectra were recorded on a confocal Raman spectrometer system (Renishaw, In Via) using a 532 nm laser as the excitation source. The magnetic measurement was carried out at room temperature by a vibrating sample magnetometer (VSM, Lakeshore-7404, USA). The complex permittivity and permeability of the composites were measured using the T/R coaxial line method in the frequency range of 2–18 GHz by a network analyzer (Agilent technologies E8362B: 10 MHz to 20 GHz). A sample containing 40 wt% of as-prepared product was pressed into a ring with an outer diameter of 7.0 mm, an inner diameter of 3.03 mm, and a length of about 3.0 mm for microwave measurement in which paraffin wax was used as the binder.

## Results and discussion

**Scheme 1:**
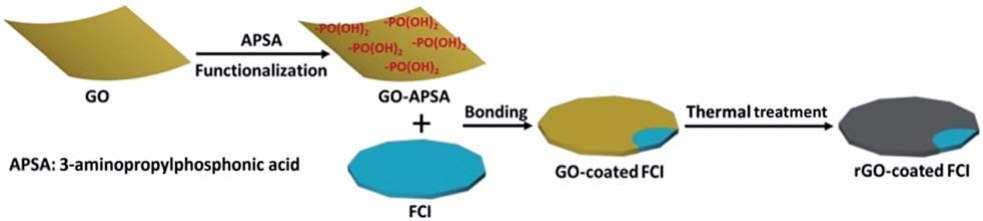
Schematic illustration of preparing rGO-coated FCI.

The strategy for synthesizing rGO-coated FCI composite is schematically depicted in Scheme 1. GO nanosheets are firstly functionalized with APSA by the reaction between amine groups of APSA and the epoxy groups of GO. Therefore, abundant –PO(OH)_2_ groups sites are located on the surface of the obtained functional GO nanosheets (GO–APSA).^38^ These –PO(OH)_2_ groups sites are highly reactive to FCI surface, which will bond with iron ions (Fe^3+^) in ultrathin surface oxide layer (Fe_2_O_3_) of FCI.^39^ From Fig. S1,† the TG curve of FCI show an obvious weight increase between 200 and 400 °C, representing the incremental rate of weight at 39.8 wt%. This is a slightly smaller than the theoretical value from FCI to Fe_2_O_3_ (42.9 wt%), suggesting that a slight oxidation should be on the surface of FCI which plays a crucial role in the successful surface coating of rGO nanosheets. Thus, when FCI encounters GO–APSA, the bonding between the surface oxide layer of FCI and –PO(OH)_2_ groups will enable GO nanosheets spontaneously coated on the surface of FCI. This designed coating between GO nanosheets and FCI will produce a so-called GO-coated FCI composite, then the residual oxygen groups were further removed and this composite will be *in situ* transformed into rGO-coated FCI composite through thermal treatment.

**Fig. 1:**
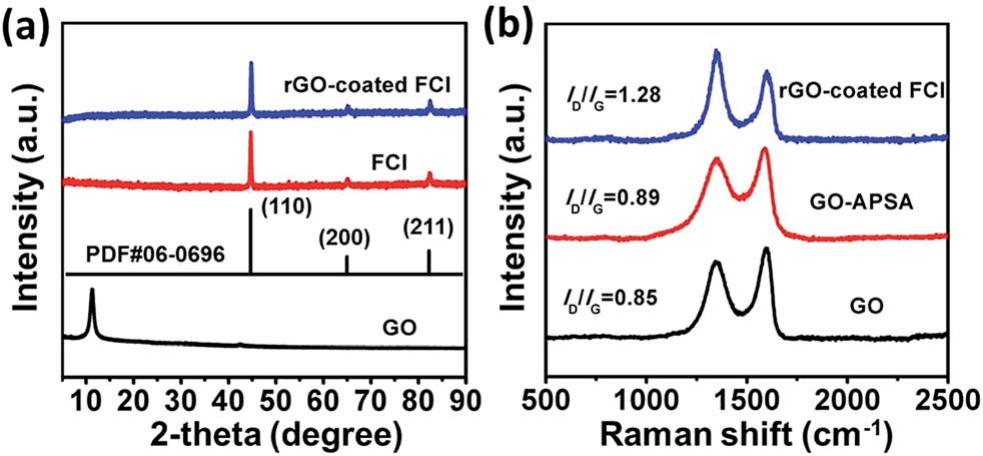
XRD patterns (a) of GO, FCI, and rGO-coated FCI; Raman spectra (b) of GO, GO–APSA, and rGO-coated FCI.

Fig. 1 shows the XRD patterns of GO, FCI, and rGO-coated FCI. GO prepared by modified Hummers method displays a characteristic peak at 11.3° corresponding to the (002) diffraction of the AB stacking order, and the presence of excessive oxygen-containing groups, such as hydroxyl, epoxy, and carboxyl groups, is always considered to be responsible for the extremely large interlayer distance (*d*-spacing) (0.78 nm).^40^ FCI exhibits three well-resolved diffraction peaks in the 2*θ* range of 5–90°, where the peaks at 2*θ* = 44.7°, 65.02°, and 82.3° can be assigned to the (110), (200), and (211) planes of iron (JCPDS no. 06-0696).^41^ The as-prepared rGO-coated FCI displays the same diffraction peaks with FCI, in which the content of rGO (∼3 wt%) is too small to show its diffraction peaks in the XRD pattern of this composite. It is well known that the bonding state of carbon atoms in carbon materials can be described by Raman spectra, and thus it can be used to analyze the evolution of carbon-based materials during synthesis process. Fig. 1b shows Raman spectra of GO, GO–APSA, and rGO-coated FCI. It can be found that these three samples all exhibit two distinguishable peaks assigned to D- and G-bands at approximately 1344 and 1580 cm^−1^, respectively, and possess different *I*_D_/*I*_G_ values. According to the exact interpretation proposed by Ferrari and Robertson,^42^ D-band is a breathing mode of A_1g_ symmetry involving phonons near the K zone boundary, which is forbidden in perfect graphite and becomes active in the presence of disorder or finite-size crystals for graphite (nanographite crystals); G-band corresponds to the E_2g_ mode due to stretching vibrations of sp^2^ bond. The ratio between the intensities of the D and G bands, *I*_D_/*I*_G_, is a useful indicator to evaluate the structural disorder of the graphene nanosheets. After the functionalization of GO with APSA, the value of *I*_D_/*I*_G_ increases slightly, from 0.85 of GO to 0.89 of GO–APSA, and the slightly higher intensity of D band suggests the presence of a bit more disorder in GO–APSA due to the bonding effect between amine groups of APSA and the epoxy groups of GO. The value of *I*_D_/*I*_G_ increases significantly from GO–APSA to rGO-coated FCI (from 0.89 to 1.28), reflecting the increase in disorder, which may be derived from the reaction between the –PO(OH)_2_ groups of GO–APSA and the FCI surface. The large increase of the value of *I*_D_/*I*_G_ indicates that the presence of located sp^3^ defects within the sp^2^ carbon network and a further increase in number of graphite crystallites. When these –PO(OH)_2_ groups of GO–APSA reacted with the ions (Fe^3+^) in ultrathin surface oxide layer (Fe_2_O_3_) of FCI, the FCI particles could adhere strongly to these chemical and physical defects of GO–APSA and the size of graphite crystallite to be smaller as well as their number to be larger.^43^ So the significant increase in the *I*_D_/*I*_G_ ratio is attributed to the decrease of the size of the ordered graphite crystallite of GO–APSA after conjugating with FCI.

**Fig. 2:**
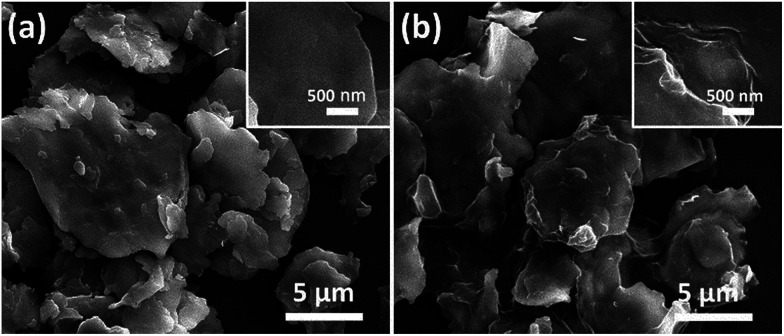
SEM images of FCI (a) and rGO-coated FCI (b).

Fig. 2 shows the morphology and microstructure of FCI and rGO-coated FCI. It is clear that the carbonyl iron in two samples both shows flaky microstructure and remain the diameter (from 2 μm to 12 μm). From a detailed observation of FCI and rGO-coated FCI, it is found that the flaky microstructure of FCI displays a smooth and clean surface (Fig. 2a, inset), whereas that of rGO-coated FCI is rough and covered with silky texture of rGO sheet (Fig. 2b, inset). SEM image in Fig. 2b shows that the FCI have been totally covered by rGO sheets, implying that bonding between FCI and –PO(OH)_2_ on the surface of GO sheets has been effectively performed. The visible wrinkles of rGO on the brink of FCI (Fig. 2b, inset) further indicates that the strong attachment between rGO nanosheets and FCI, and this strong attachment is not only beneficial to produce sufficient synergetic behaviors and interfacial effects, but also is helpful to achieve the charge transfer at the interface of FCI and rGO nanosheets.^44^

**Fig. 3:**
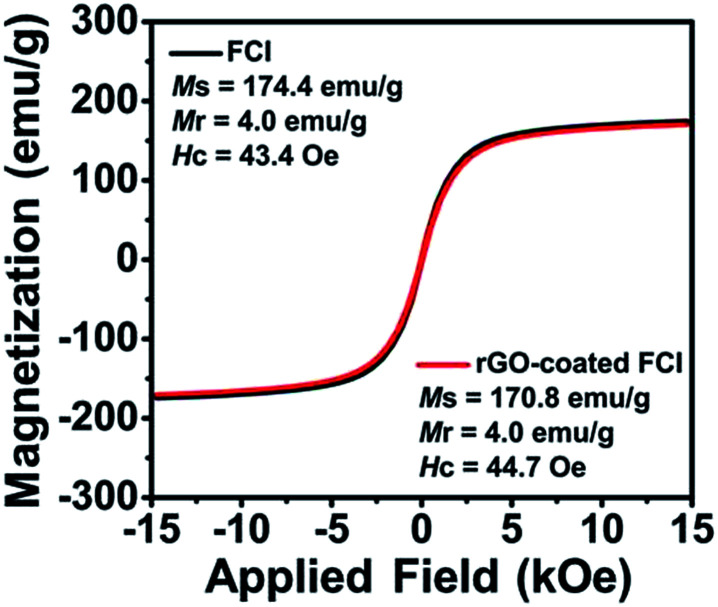
Magnetization hysteresis loops of FCI and rGO-coated FCI.

Fig. 3 shows the hysteresis loops of FCI and rGO-coated FCI, which both exhibit ferromagnetic characteristics. Although the saturation magnetization (*M*_s_) of the rGO-coated FCI was reduced slightly compared to that of FCI, which is believed to have been caused by the non- or low-magnetic GO shell coating. These two samples all present very strong saturation magnetization (*M*_s_) at 174.4 and 170.8 emu g^−1^, respectively, and their intrinsic coercivity (*H*_c_) and remanent magnetization (*M*_r_) are also similar. The higher *M*_s_ and lower *H*_c_ are beneficial to the improvement of the *μ*_r_ value, which can improve the microwave absorption properties. It can be indicated that less content of antiferromagnetic rGO nanosheets (∼3%) on the surface of FCI make less influence on the magnetic properties of FCI. In our case, magnetic properties of FCI and rGO-coated FCI are very close, implying their similarly initial permeability.

**Fig. 4:**
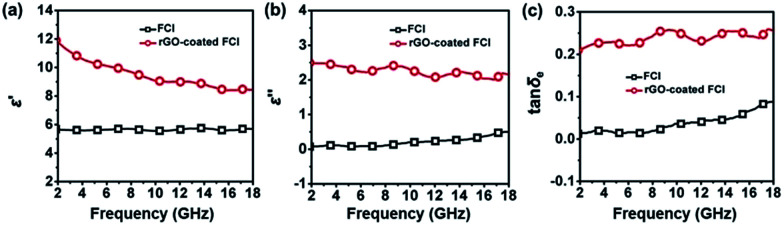
Real parts (a) and imaginary parts (b) of the complex permittivity, and corresponding dielectric loss tangents (c) of FCI.

Complex permittivity (*ε*_r_ = *ε*′ − *jε*′′) and complex permeability (*μ*_r_ = *μ*′ − *jμ*′′) are very important parameters that can determine the microwave absorption properties of an absorber, where the real parts of complex permittivity (*ε*′) and complex permeability (*μ*′) represent the storage capability of electric and magnetic energy, and the imaginary parts (*ε*′′) and (*μ*′′) describe the loss capability of electric and magnetic energy.^45^Fig. 4a and b show the complex permittivity of FCI and rGO-coated FCI in the frequency range of 2.0–18.0 GHz. *ε*′ values of FCI are almost constant in the whole frequency range and *ε*′′ values of FCI slightly increase from 0.08 at 2.0 GHz to 0.50 at 18.0 GHz. By comparison, rGO-coated FCI possess much higher real and imaginary parts of complex permittivity and shows a typical frequency dispersion behavior, whose *ε*′ values decrease from 11.83 at 2 GHz to 8.44 at 18.0 GHz. Typical frequency dispersion behavior of *ε*′ is always greatly beneficial to the characteristic impedance match in magnetic metal-based composite microwave absorbers.^46^ And the typical frequency dispersion behavior of *ε*′ discovered in rGO-coated FCI composite will also contribute to its characteristic impedance match as microwave absorber which will be discussed in the latter part of this article. It is worth noting that this frequency dispersion behavior comes from the introduction of rGO and its complete surface coating to FCI. According to the first principles, charge transfer would occur between metal and graphene through the direct metal–graphene interaction.^44^ This theoretical prediction has been demonstrated in a Fe–graphene heterostructure, in which the electron transfer from Fe to graphene is observed.^47^ In our case, a charge transfer from FCI to rGO can be reasonably assumed in the interface of FCI and rGO, leading to the introduction of free charge into rGO. The introduced free charge would vibrate with the stimuli of the micro and give rise to the electric polarization in rGO, which increase the value of *ε*′. Furthermore, the value of *ε*′ would decrease with the increase of the frequency due to the relaxation of polarization, showing a dielectric dispersion.^48^ Meanwhile, the motion of the introduced free charge would attenuate the microwave energy, resulting in the enhancement of the dielectric loss. To verify this, a control composite, rGO/FCI, is prepared by ultrasonic mixing GO nanosheets and FCI by same chemical composition in the preparation of rGO-coated FCI and subsequently performing a same thermal reduction treatment which has been used in the synthesis process of rGO-coated FCI. The control composite displays completely different microstructure from rGO-coated FCI, in which the rGO nanosheets aggregate together and the external surface of FCI has been covered by nothing (Fig. S2†). The complex permittivity of rGO/FCI (Fig. S3a†) has not shown the typical dielectric dispersion behavior and the values of complex permittivity in the whole applied frequency are also smaller than those of rGO-coated FCI. The charge transfer could not occur because there is no interaction between the FCI and rGO in rGO/FCI. The dielectric loss tangents (tan *δ*_e_ = *ε*′′/*ε*′), a concept that is normally used to evaluate dielectric loss ability, further confirm that rGO-coated FCI has larger dielectric loss ability than FCI and rGO/FCI (Fig. 4c and S3c†), indicating the superiority of rGO-coated FCI composite in microwave absorption. These may be due to that the uneven distribution of space charges between rGO nanosheets and FCI will bring additional interfacial polarization for rGO-coated FCI, and meanwhile, the defects and residual groups in rGO nanosheets can also accumulate abundant bound charges to generate substantial orientation polarization.

**Fig. 5:**
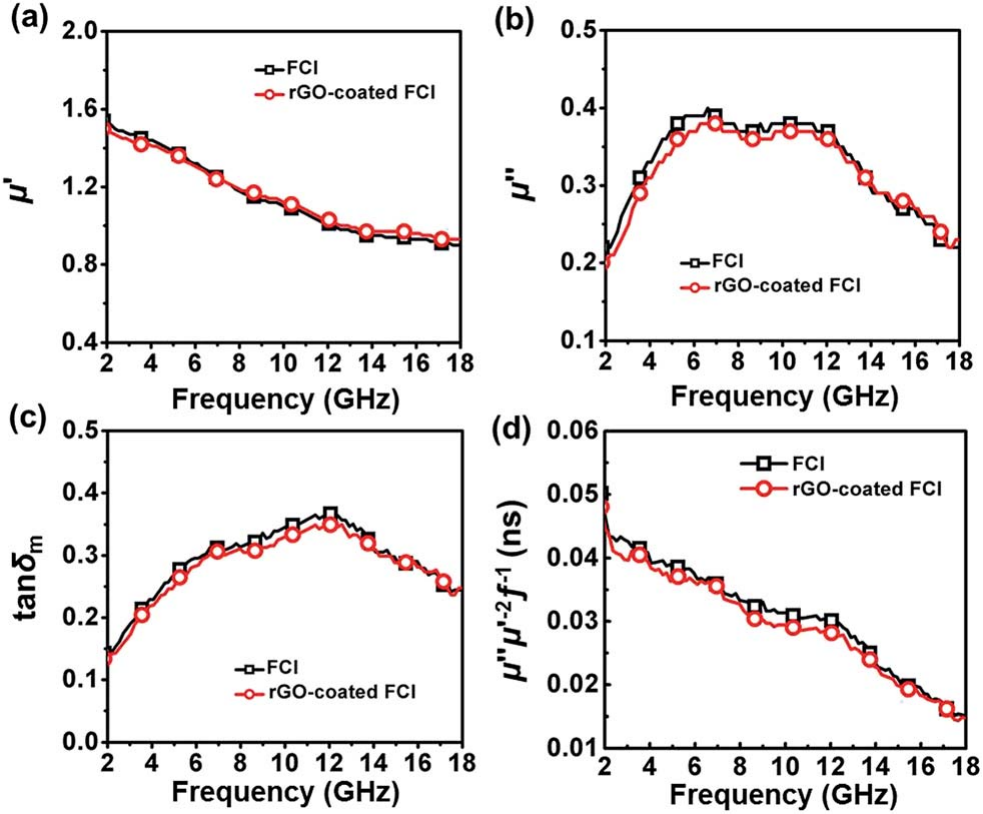
Real parts (a) and imaginary parts (b) of the complex permeability, corresponding magnetic loss tangents (c) and the value of *μ*′′*μ*′^−2^*f*^−1^ (d) of the two samples.

Fig. 5a–c show the real parts and imaginary parts of complex permeability and corresponding magnetic loss tangents tan *δ*_m_ = *μ*′′/*μ*′, a concept that is normally used to evaluate magnetic loss ability of FCI and rGO-coated FCI composite in the frequency range of 2.0–18.0 GHz. It is shown that the variations of the complex permeability and magnetic loss tangents for FCI and rGO-coated FCI are very similar, suggesting that the magnetic loss mechanisms of FCI and rGO-coated FCI are almost identical. In gigahertz range, natural ferromagnetic resonance and eddy current effect usually could be the main loss mechanisms for ferromagnetic absorbers.^49^ The eddy current loss can be expressed by^49^

1 *μ*′′ = 2π*μ*_0_(*μ*′)^2^*σd*^2^*f*/3

where *σ* (S m^−1^) is the electrical conductivity and *μ*_0_ (H m^−1^) is the permeability in vacuum. If the reflection loss only comes from the eddy current effect, the value of *μ*′′*μ*′^−2^*f*^−1^ will be constant when the frequency changes.^49^Fig. 5d demonstrates that the values of *μ*′′*μ*′^−2^*f*^−1^ for FCI and rGO-coated FCI both continuously change from 2.0 to 18.0 GHz, suggesting the rather limited impact from the eddy current loss. Therefore, natural ferromagnetic resonance should be the main magnetic loss mechanisms both for FCI and rGO-coated FCI.

**Fig. 6:**
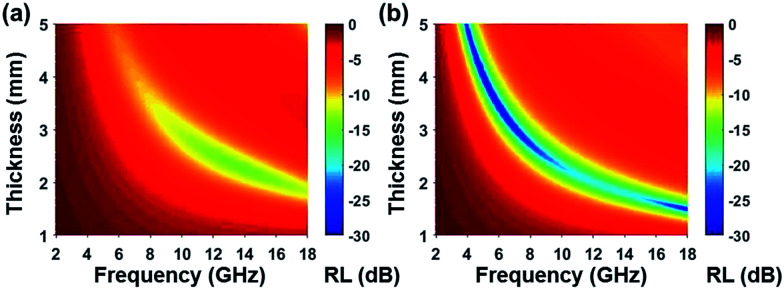
Reflection loss mapping of FCI (a) and rGO-coated FCI with absorbers thickness from 1 mm to 5 mm in the frequency range of 2.0–18.0 GHz.

Based on complex permittivity and complex permeability, Fig. 6 presents a comparison of calculated reflection loss properties (*R*_L_ (dB)) of FCI and rGO-coated FCI in the frequency range of 2.0–18.0 GHz with varying absorber thickness (*d*) from 1.0 to 5.0 mm. These two samples both can attenuate incident electromagnetic waves, but their performances present significant difference. For example, FCI only gives unimpressive reflection loss properties in the studied frequency range with a minimum value of −13.8 dB at 13.7 GHz at an absorber thickness of 2.28 mm. Compared with FCI, rGO-coated FCI represents an excellent microwave absorbing performance with strong reflection loss (−65.4 dB at 5.2 GHz) when absorber thickness is 3.87 mm. The reflection loss map in Fig. 6b is artificially truncated to −30.0 dB for clarity, and the corresponding reflection loss curves can be found in Fig. S4.† Furthermore the reflection loss of rGO-coated FCI shows effective bandwidth under −20 dB when absorber thickness is from 1.5 mm to 5 mm. For the control composite of rGO/FCI, its minimum value of reflection loss is −16.69 dB at 12.4 GHz with an absorber thickness of 2.32 mm (Fig. S3d†), which is relatively inferior to the performance of rGO-coated FCI, further indicating the superiority of surface coating of rGO nanosheets on FCI. Although the complex permeability of the FCI, rGO-coated FCI and rGO/FCI are similar, the complex permittivity and characteristic impedance lead to the different reflection loss properties.

Characteristic impedance is also an important factor which can determine the microwave absorption performance of microwave absorbing materials. The characteristic impedance of the absorbing materials should be equal/close to that of the free space (377 Ω sq^−1^) to achieve zero reflection at the front surface of the materials. Specifically, the matched characteristic impedance is always determined by the relationship between the complex permittivity and complex permeability. A delta-function method is usually used to validate the impedance matching degree by the following equation,^18^

2 |*Δ*| = |sinh^2^(*Kfd*) − *M*|

where *K* and *M* are determined by the complex permittivity and permeability in the following equations3
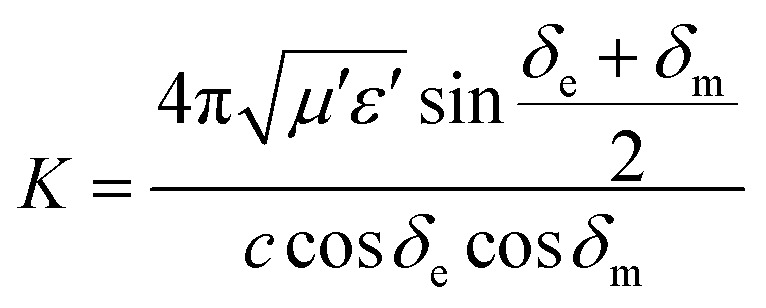
4
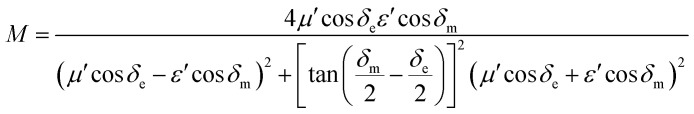


**Fig. 7:**
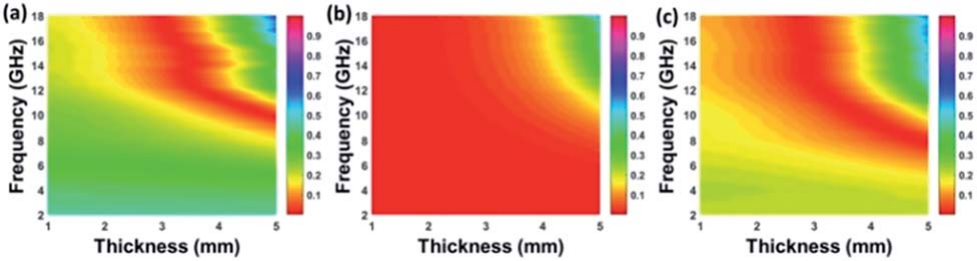
Calculated delta value maps of FCI (a), rGO-coated FCI composite (b) and the control composite of rGO/FCI (c).

According to those equations, a smaller delta value implies better impedance matching. Fig. 7 shows the calculated delta value maps of FCI, rGO-coated FCI and rGO/FCI. Obviously, rGO-coated FCI have larger area close to zero, which can directly explain better matching of characteristic impedance in rGO-coated FCI, although the values of complex permittivity in rGO-coated FCI in the whole applied field are higher than the corresponding values in FCI and rGO/FCI, respectively. Therefore, it can be speculated that it is the typical frequency dispersion behavior of complex permittivity in rGO-coated FCI which contributes to the characteristic impedance matching, because its complex permeability also exhibits typical frequency dispersion behavior.

## Conclusions

We demonstrate here the construction of rGO-coated FCI composite, presenting complete surface coating of rGO nanosheets on FCI. The complex permittivity of rGO-coated FCI displays typical frequency dispersion behavior which is demonstrated to be very beneficial to improve the characteristic impedance matching through a delta-function method, further verifying the superiority of this composite. This composite also shows excellent dielectric loss which makes contribution to its microwave absorption performance. Comparing to the control composite of rGO/FCI, the homogeneous microstructure of rGO-coated FCI plays a crucial role to its excellent microwave absorption performance. The rGO-coated FCI composite gives a minimum value of reflection loss with −65.4 dB at 5.2 GHz when the absorber thickness is 3.87 mm and shows effective bandwidth under −20 dB when absorber thickness is from 1.5 mm to 5 mm.

## Conflicts of interest

There are no conflicts to declare.

## Supplementary Material

RA-008-C7RA12984J-s001

## References

[cit1] Wang Y., Du Y. C., Xu P., Qiang R., Han X. J. (2017). Polymers.

[cit2] Sun G. B., Dong B. X., Cao M. H., Wei B. Q., Hu C. W. (2011). Chem. Mater..

[cit3] Shi X. L., Cao M. S., Yuan J., Fang X. Y. (2009). Appl. Phys. Lett..

[cit4] Wang C., Han X. J., Xu P., Wang J. Y., Du Y. C., Wang X. H., Qin W., Zhang T. (2010). J. Phys. Chem. C.

[cit5] Zhu W. M., Wang L., Zhao R., Ren J. W., Lu G. Z., Wang Y. Q. (2011). Nanoscale.

[cit6] Ni S. B., Lin S. M., Pan Q. T., Yang F., Huang K., He D. Y. (2009). J. Phys. D: Appl. Phys..

[cit7] Du Y. C., Wang J. Y., Cui C. K., Liu X. R., Wang X. H., Han X. J. (2010). Synth. Met..

[cit8] Wu R. B., Zhou K., Yang Z. H., Qian X. K., Wei J., Liu L., Huang Y. Z., Kong L. B., Wang L. Y. (2013). CrystEngComm.

[cit9] Zhu Y. F., Zhang L., Natsuki T., Fu Y. Q., Ni Q. Q. (2012). ACS Appl. Mater. Interfaces.

[cit10] Zhang P., Han X. J., Kang L. L., Qiang R., Liu W. W., Du Y. C. (2013). RSC Adv..

[cit11] Che R. C., Peng L. M., Duan X. F., Chen Q., Liang X. L. (2004). Adv. Mater..

[cit12] Wang Y. F., Chen D. L., Yin X., Xu P., Wu F., He M. (2015). ACS Appl. Mater. Interfaces.

[cit13] Li Y. N., Zhao Y., Lu X. Y., Zhu Y., Jiang L. (2016). Nano Res..

[cit14] Han R., Qiao L. A., Wang T., Li F. S. (2011). J. Alloys Compd..

[cit15] Wang A. M., Wang W., Long C., Li W., Guan J. G., Gu H. S., Xu G. X. (2014). J. Mater. Chem. C.

[cit16] Zheng D. L., Liu T., Zhou L., Xu Y. G. (2016). J. Magn. Magn. Mater..

[cit17] Zhang W. Q., Bie S. W., Chen H. C., Lu Y., Jiang J. J. (2014). J. Magn. Magn. Mater..

[cit18] Ma Z., Zhang Y., Cao C. T., Yuan J., Liu Q. F., Wang J. B. (2011). Phys. B.

[cit19] Sui M. X., Lu X. L., Xie A., Xu W. D., Rong X. H., Wu G. J. (2015). Synth. Met..

[cit20] Liao L., Lin Y. C., Bao M. Q., Cheng R., Bai J. W., Liu Y. A., Qu Y. Q., Wang K. L., Huang Y., Duan X. F. (2010). Nature.

[cit21] Bae S., Kim H., Lee Y., Xu X. F., Park J. S., Zheng Y., Balakrishnan J., Lei T., Kim H. R., Song Y. I., Kim Y. J., Kim K. S., Ozyilmaz B., Ahn J. H., Hong B. H., Iijima S. (2010). Nat. Nanotechnol..

[cit22] Gwon H., Kim H. S., Lee K. U., Seo D. H., Park Y. C., Lee Y. S., Ahn B. T., Kang K. (2011). Energy Environ. Sci..

[cit23] Yavari F., Koratkar N. (2012). J. Phys. Chem. Lett..

[cit24] Wang C., Han X. J., Xu P., Zhang X. L., Du Y. C., Hu S. R., Wang J. Y., Wang X. H. (2011). Appl. Phys. Lett..

[cit25] Zhang Y., Huang Y., Chen H. H., Huang Z. Y., Yang Y., Xiao P. S., Zhou Y., Chen Y. S. (2016). Carbon.

[cit26] Zhao B., Fan B. B., Shao G., Zhao W. Y., Zhang R. (2015). ACS Appl. Mater. Interfaces.

[cit27] Wang Y., Du Y. C., Qiang R., Tian C. H., Xu P., Han X. J. (2016). Adv. Mater. Interfaces.

[cit28] Qing Y. C., Nan H. Y., Luo F., Zhou W. C. (2017). RSC Adv..

[cit29] Tadjarodi A., Rahimi R., Imani M., Kerdari H., Rabbani M. (2012). J. Alloys Compd..

[cit30] Liu P. B., Huang Y., Zhang X. (2014). J. Alloys Compd..

[cit31] Liu P. B., Huang Y., Wang L., Zong M., Zhang W. (2013). Mater. Lett..

[cit32] Sun C., Jiang W., Wang Y. J., Sun D. P., Liu J., Li P. Y., Li F. S. (2014). Phys. Status Solidi RRL.

[cit33] Qing Y. C., Min D. D., Zhou Y. Y., Luo F., Zhou W. C. (2015). Carbon.

[cit34] Zhu Z. T., Sun X., Xue H. R., Guo H., Fan X. L., Pan X. C., He J. P. (2014). J. Mater. Chem. C.

[cit35] Xu Y., Luo J. H., Yao W., Xu J. G., Li T. (2015). J. Alloys Compd..

[cit36] Zhang W. L., Choi H. J. (2014). J. Appl. Phys..

[cit37] Schniepp H. C., Li J. L., McAllister M. J., Sai H., Herrera-Alonso M., Adamson D. H., Prud'homme R. K., Car R., Saville D. A., Aksay I. A. (2006). J. Phys. Chem. B.

[cit38] He L. H., Zhao Y., Xing L. Y., Liu P. G., Wang Z. Y., Zhang Y. W., Liu X. F. (2017). Appl. Surf. Sci..

[cit39] Yee C., Kataby G., Ulman A., Prozorov T., White H., King A., Rafailovich M., Sokolov J., Gedanken A. (1999). Langmuir.

[cit40] Liu J. Q., Tang J. G., Gooding J. J. (2012). J. Mater. Chem..

[cit41] Feng Y. B., Qiu T., Shen C. Y. (2007). J. Magn. Magn. Mater..

[cit42] Ferrari A. C., Robertson J. (2000). Phys. Rev. B.

[cit43] Sun X., He J. P., Li G. X., Tang J., Wang T., Guo Y. X., Xue H. R. (2013). J. Mater. Chem. C.

[cit44] Giovannetti G., Khomyakov P. A., Brocks G., Karpan V. M., van den Brink J., Kelly P. J. (2008). Phys. Rev. Lett..

[cit45] Chen N., Mu G. H., Pan X. F., Gan K. K., Gu M. Y. (2007). Mater. Sci. Eng., B.

[cit46] Zhao X. C., Zhang Z. M., Wang L. Y., Xi K., Cao Q. Q., Wang D. H., Yang Y., Du Y. W. (2013). Sci. Rep..

[cit47] Pi K., McCreary K. M., Bao W., Han W., Chiang Y. F., Li Y., Tsai S. W., Lau C. N., Kawakami R. K. (2009). Phys. Rev. B.

[cit48] Sameshima T., Hayasaka H., Haba T. (2009). Jpn. J. Appl. Phys..

[cit49] Hou Z. L., Zhou H. F., Kong L. B., Jin H. B., Qi X., Cao M. S. (2012). Mater. Lett..

