# Supplementary material for: Preparation of reduced graphene oxide coated flaky carbonyl iron composites and their excellent microwave absorption properties
Source: RSC Adv. 2018 Jan 15;8(6):2971–7. doi: 10.1039/c7ra12984j (PMC9077548; doi:10.1039/c7ra12984j)
Supplement: RA-008-C7RA12984J-s001 [file RA-008-C7RA12984J-s001.pdf]

Electronic Supplementary Material (ESI) for RSC Advances.
This journal is © The Royal Society of Chemistry 2018

Supplementary information for:

Preparation of reduced graphene oxide coated flaky carbonyl iron composites and their excellent microwave absorption properties

Lihua He[a,b], Yan Zhao[a,*], Liying Xing[b], Pinggui Liu[b], Zhiyong Wang[b], Youwei Zhang[b], Ying Wang[c], Yunchen Du[c]

[a] School of Materials Science and Engineering, Beihang University, Beijing 100191, China.

[b] Beijing Institute of Aeronautical Materials, Beijing 100095, China.

[c] MIIT Key Laboratory of Critical Materials Technology for New Energy Conversion and Storage, School of Chemistry and Chemical Engineering, Harbin Institute of Technology, Harbin 150001, China

Corresponding author: Yan Zhao. Tel/Fax:+86-010-82317127.

E-mail address: Helihua-xjt@163.com;jennyzhaoyan@buaa.edu.cn

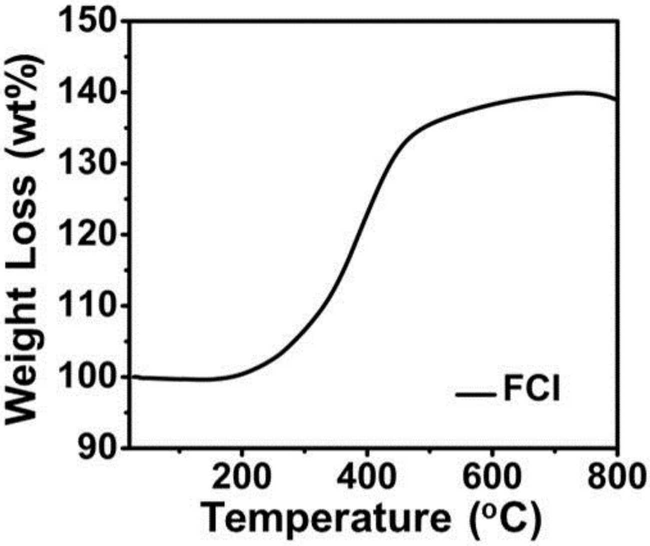

Fig. S1. TG curves of FCI.

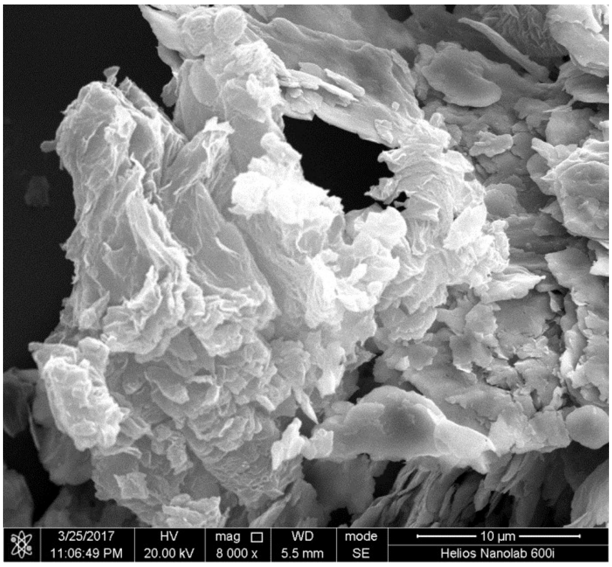

Fig. S2. SEM image of control composite of rGO/FCI.

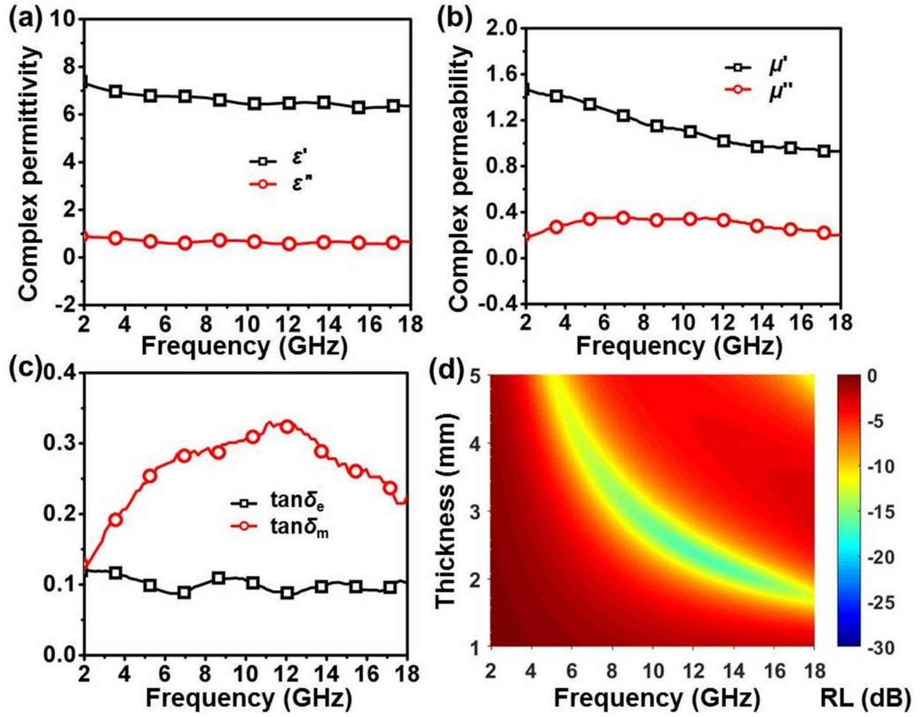

Fig. S3. Complex permittivity (a), complex permeability (b), dielectric loss tangent and magnetic loss tangent (c) and reflection loss of rGO/FCI composite in the frequency range of 2.0-18.0 GHz.

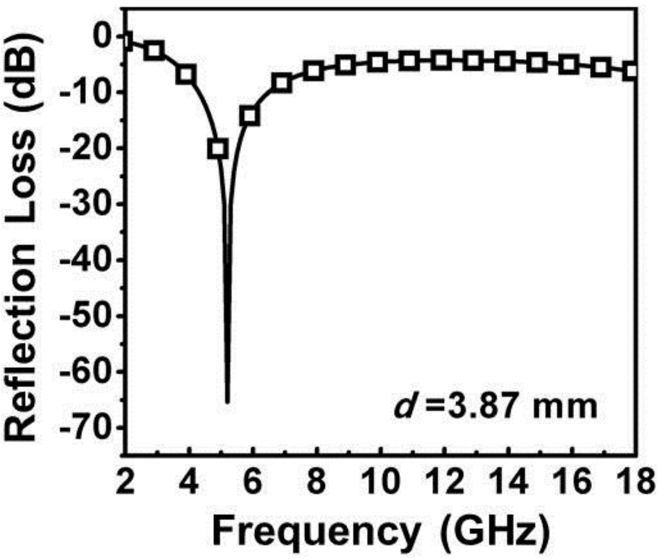

Fig. S4. Reflection loss of rGO-coated FCI composite in the frequency range of 2.0-18.0 GHz with an absorber thickness of 3.87 mm.
